# Treatment with Y-27632, a ROCK Inhibitor, Increases the Proinvasive Nature of SW620 Cells on 3D Collagen Type 1 Matrix

**DOI:** 10.1155/2012/259142

**Published:** 2012-05-29

**Authors:** Ramana Vishnubhotla, Shruthi Bharadwaj, Shan Sun, Vitali Metlushko, Sarah C. Glover

**Affiliations:** ^1^Department of Bioengineering, University of Illinois at Chicago, Chicago, IL 60612, USA; ^2^Department of Medicine, University of Florida, P.O. Box 100214, Gainesville, FL 32610, USA; ^3^Department of Biomedical Engineering, University of Florida, Gainesville, FL 32610, USA; ^4^Department of Electrical and Computer Engineering, University of Illinois at Chicago, Chicago, IL 60607, USA

## Abstract

The concept of using tissue density as a mechanism to diagnose a tumor has been around for centuries. However, this concept has not been sufficiently explored in a laboratory setting. Therefore, in this paper, we observed the effects of cell density and extracellular matrix (ECM) density on colon cancer invasion and proliferation using SW620 cells. We also attempted to inhibit ROCK-I to determine its effect on cell invasion and proliferation using standard molecular biology techniques and advanced imaging. Increasing cell seeding density resulted in a 2-fold increase in cell invasion as well as cell proliferation independent of treatment with Y-27632. Increasing collagen I scaffold density resulted in a 2.5-fold increase in cell proliferation while treatment with Y-27632 attenuated this effect although 1.5 fold increase in cell invasion was observed in ROCK inhibited samples. Intriguingly, ROCK inhibition also resulted in a 3.5-fold increase in cell invasion within 3D collagen scaffolds for cells seeded at lower densities. We show in this paper that ROCK-I inhibition leads to increased invasion within 3D collagen I microenvironments. This data suggests that although ROCK inhibitors have been used clinically to treat several medical conditions, its effect largely depends on the surrounding microenvironment.

## 1. Introduction

Colon cancer is the third most commonly diagnosed cancer and the third leading cause of cancer death in both men and women in the USA [1, 2]. Today, there is a wide array of methods used to diagnose cancer including biopsy, endoscopy, and diagnostic imaging. Imaging techniques utilize the fact that tumorigenic tissue has a higher tissue density than the surrounding normal extracellular matrix (ECM). Thus, areas of increased tissue density are considered a warning sign of a potential malignancy [3–5]. With this strong link between tissue density and cancer, there has not been sufficient *in vitro* data, particularly for colon cancer, to fully understand this phenomenon.

Two variables that affect mechanics of a tissue are cell and ECM density. Altering cell density induces cellular differentiation, proliferation, and even apoptosis [6, 7]; thus, cell density is one of the relevant parameters in cancer research. Previous studies have suggested that higher cell density environments significantly increase cell metastasis, especially colon 26, [8] and the initial seeding density affects differentiation of stem cells more than the cytokines and growth factors [9].

Similarly, mechanical induction done by altering the surrounding ECM alone affects cellular differentiation, proliferation, and apoptosis [10, 11]. This can be attributed to mechanical cues, which affect cytoskeletal arrangement through Rho-kinase (ROCK). It has been shown in the literature that ROCK is, in fact, responsible for regulating morphology of cells by altering actin cytoskeleton [12]. Also, activation of ROCK promotes force generation that contributes to various cell processes such as cell motility and adhesion [13]. The two isoforms of ROCK, ROCK-1, and ROCK-II, have been shown to express similar phenotypes [14] although their cellular localization is different [15, 16]. The modulators that activate or repress the two isoforms of ROCK are also different [13, 17]. These differences may be responsible for the distinctive functions of ROCK-I and ROCK-II within the cell. Literature suggests that ROCK-I knockdown promotes keratinocyte terminal differentiation, whereas ROCK-II knockdown inhibits keratinocyte terminal differentiation [18]. Y-27632 is a highly potent, cell-permeable, selective inhibitor of Rho-associated protein kinases [19].

We have previously shown that ROCK localizes to invadopodia in colon cancer where it appears to regulate the activities of MMP-2 and MMP-13 [20]. These experiments were performed in 1.5 mg/mL scaffolds. However, tumor tissue typically has a rigidity consistent with collagen I concentrations much greater than 2 mg/mL [21]. While the scaffolds used in the previous study were of significant density, they were not as dense as tissue that might be found in a tumor. Therefore, the aim of this study was to define how both tissue and cell density impact ROCK-1-mediated proliferation and invasion.

## 2. Materials and Methods

### 2.1. Reagents and Supplies

All cell culture reagents, excluding FBS (Gemini Bio-products), were obtained from Mediatech, Inc. (Herndon, VA). SW620 cells (ATCC # CCL-227) were purchased from ATCC (Manassas, VA). Cells were maintained in dishware from BD Falcon (Lincoln Park, NJ). Type I rat tail collagen was purchased from BD Bioscience (Bedford, MA). Mammalian protease inhibitor cocktail was purchased from Sigma-Aldrich (St. Louis, MO). Cell extracts were equalized using the BCA Protein Assay Kit from Pierce (Rockford, IL). SiRNA against ROCK-I (sc-29473) was purchased from Santa Cruz Biotechnology (Santa Cruz, CA). *Silencer* siRNA Transfection II Kit was purchased from Invitrogen (Eugene, OR). Rho-kinase activity was measured using a Rho-kinase assay kit available from Cyclex (Nagano, Japan). Proliferation was observed using “Vybrant MTT Cell Proliferation Assay Kit” from Invitrogen (Carlsbad, CA). CellTracker Orange CMTMR was purchased from Invitrogen (Eugene, OR). Colorimetric invasion assays were purchased from Millipore (Bellerica, MA). All other supplies were molecular biology grade and were from Fisher (Pittsburg, PA).

### 2.2. Cell Culture

SW620 cells were cultured in Leibovitz's L-15/10% FBS containing 2 mM L-Glutamine and were incubated at 37°C in a 0% CO_2_ humidified environment.

### 2.3. Preparation of 3D Scaffolds

Three-dimensional (3D) scaffolds were constructed using type I collagen-based hydrogels. Briefly, 9 mg/mL type I collagen in 0.1 M acetic acid was neutralized to a pH of 7.4 with of 1.0 M NaOH in PBS 10X for a final type I collagen concentration of 1.5 or 4.0 mg/mL. Predetermined amounts of this mixture were placed in wells and allowed to polymerize. SW620 cells were plated at seeding densities of 50 × 10^3^ and 250 × 10^3^ cells/cm^2^ on each well. Gels were incubated at 37°C for 1 hr. at which time an additional 2 mL of appropriate media was added.

 SW620 cells were seeded at 50 and 250 × 10^3^ cells/cm^2^ onto 1.5 or 4 mg/mL collagen I gels and incubated at 37°C for a period of five days. Scaffolds were treated with 10 *μ*M of the ROCK inhibitor Y-27632 added daily to the scaffolds. No treatment with ROCK inhibitor was used as the control condition.

### 2.4. Multiphoton Microscopy

 Cell-containing collagen scaffolds were cultured for up to 5 days in a 37°C incubator (no CO_2_). Scaffolds were then washed with phosphate buffered saline (PBS), fixed overnight with 10% neutral buffered formalin (NBF), and then permeabilized with Tris-buffered saline with 0.05% Tween-20 (TBST) for 15 minutes. After this, the scaffolds were blocked for 20 minutes with serum-free protein block and then washed with TBST. Scaffolds were labeled with 10 *μ*M of a CellTracker Orange CMTMR dye for 30 minutes and then washed with PBS.

 Seeded cells were imaged by a laser scanning multiphoton confocal microscope with 40x objective (NA = 1.40). Cells labeled with the CellTracker dye were visualized by using multiphoton laser excitation at 541 nm and emission at 565 nm, for which the femotsecond laser beam (80 MHz, 0.5 mW), pumped from a mode-locked titanium:Sapphire laser (MaiTai, Spectra-Physics Inc., CA), was coupled with visible laser (Bio-Rad, UK) into an inverted laser scanning confocal microscope (Nikon TE200-U, Japan).

### 2.5. Boyden Chamber Assay

 Initial cell invasion was analyzed using a commercially available colorimetric invasion assay. The assay consists of inserts with 8 *μ*m pore polycarbonate membrane precoated with collagen. The inserts were soaked in warm serum-free media in order to rehydrate the collagen coating. Next, SW620 cell suspensions were created and added to the inserts in order to give seeding densities of 50 × 10^3^ or 250 × 10^3^ cells/cm^2^ in each insert. Media supplemented with 10% FBS was added to the bottom chamber, making sure the membrane has full contact with the media in the lower chamber.

The wells were then incubated at 37°C for 72 hours in 0% CO_2_ humidified environment. After 3 days, media was removed and the insert containing the membrane was placed in wells containing a cell stain. The inserts were washed in water and noninvading cells were removed from the top portion of the insert using a clean cotton swab. The inserts were then placed extraction buffer and following extraction, an absorbance reading was taken at 550 nm. See proliferation assay for details.

### 2.6. Proliferation Assay

 Cell proliferation was determined using a standard MTT assay kit. To do this, the media were removed from each chamber and the gels were washed with PBS. Fresh L-15 media without phenol red and MTT dye, ((3-(4,5-dimethylthiazol-2-yl)-2,5-diphenyltetrazolium bromide), in PBS were added to each chamber in a 5 : 1 ratio. The gels were incubated at 37°C for four hours. After four hours, supernatant from each chamber was combined with dimethyl sulfoxide (DMSO) in a 1 : 2 ratio. Absorbance was read at 550 nm.

### 2.7. ROCK Activity

In order to reach the desired seeding density, cells were plated at a density of 50 × 10^3^ cells/cm^2^ in 6-well plates and 250 × 10^3^ cells/cm^2^ in 24-well plates. Cells were treated with 10 *μ*M Y-27632, a common ROCK inhibitor and the untreated condition was used as the control.

ROCK activity was analyzed using an ELISA kit. Briefly, each sample in kinase reaction buffer (1 : 10 ratio) was added to each precoated well. After washing, each well was incubated with an HRP-conjugated antibody for one hour after which a detection solution was added to detect the presence of the antibody. A dual absorbance measurement was taken at 450/550 nm using a standard microplate reader.

### 2.8. Transfection with SiRNA

SW620 cells were transfected with siRNA to knockdown ROCK-I, where untransfected cells were used as the control. After transfection, the cells were seeded onto collagen I gels of concentrations of 2.0 and 4.0 mg/mL and incubated at 37°C for a period of three days.

### 2.9. Statistics

Data was analyzed using Student's *t*-distribution using average values and the associated standard deviation. A comparative *P* value of less than 0.05 was considered significant.

## 3. Results

### 3.1. Cell Seeding Density Affects Characteristics in 3D

**Figure 1:**
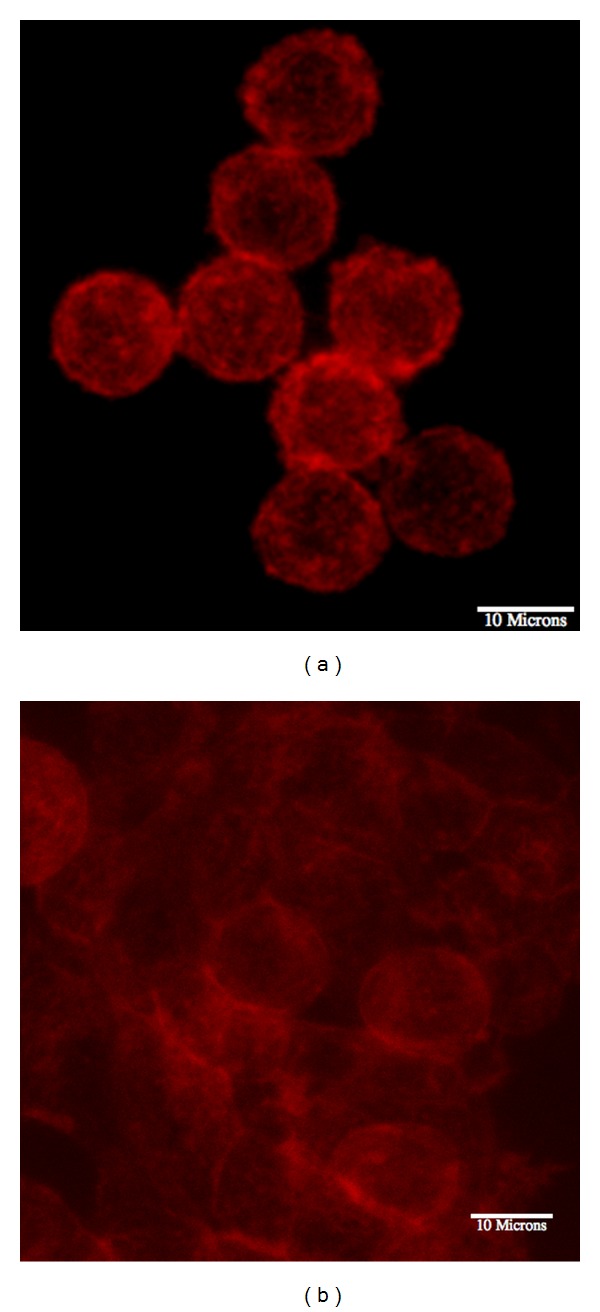
SW620 cells were seeded at different densities: 50 × 10^3^ (a), and 250 × 10^3^ cells/cm^2^ (b). The lower seeding density (a) shows rounder cells with few cell-cell contacts. The greater seeding density allows for more cell-cell contact, creating a more epithelial state. Scale Bar = 10 *μ*m.

Collagen scaffolds were prepared, and SW620 cells were seeded as described in the methods section. Cells were stained with phalloidin and imaged using a multiphoton microscope. Cells seeded at the lower cell density appeared to have relatively few cell-cell interactions. Within the collagen scaffold, these cells exhibited a symmetrically round phenotype. No protrusions or invadopodia were as observed in prior experiments using SW620 cells in 1.5 mg/mL scaffolds [20]. Cells seeded at the higher density, 4 mg/mL, were tightly packed within the 3D scaffold resulting in a forced cell-to-cell contact. It is important to note that the cells that appear “rounder” are on a plane above the rest of the cells. This can be seen in Figure 1.

### 3.2. Increased Cell Density Leads to an Increase in ROCK Activity

**Figure 2:**
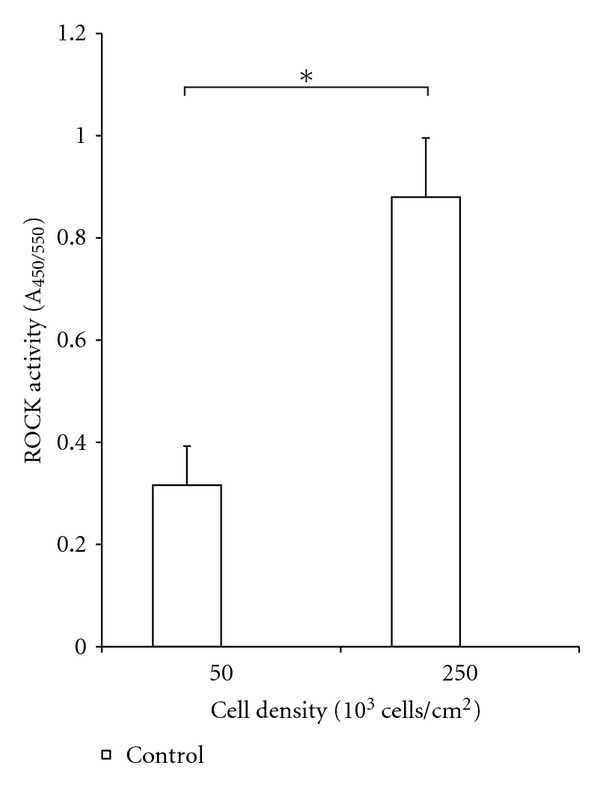
ROCK activities of SW620 cells seeded at 50 × 10^3^ and 250 × 10^3^ cells/cm^2^ and allowed to invade into 1.5 mg/mL collagen scaffolds. Cells seeded at the higher density had over double the ROCK activity compared to those seeded at the lower density. The number of cells was kept constant between the two conditions while the seeding area was altered.

 ROCK activity was significantly increased with an increase in cell density. A 2.5-fold increase in ROCK activity was observed in scaffolds with cells seeded at 250 × 10^3^ cells/cm^2^ compared to scaffold with cells seeded at 50 × 10^3^ cells/cm^2^. It is important to note that cell density was altered by changing the seeding area and keeping the number of cells seeded constant. This was done to determine the effect that cell density alone, and not the number of cells, has on ROCK activity. Standard Student *t*-test was performed and a *P* value of 0.05 was considered significant (∗). The data is shown in Figure 2.

### 3.3. Treatment with Y-27632 Decreases Invasion in Boyden Chambers

**Figure 3:**
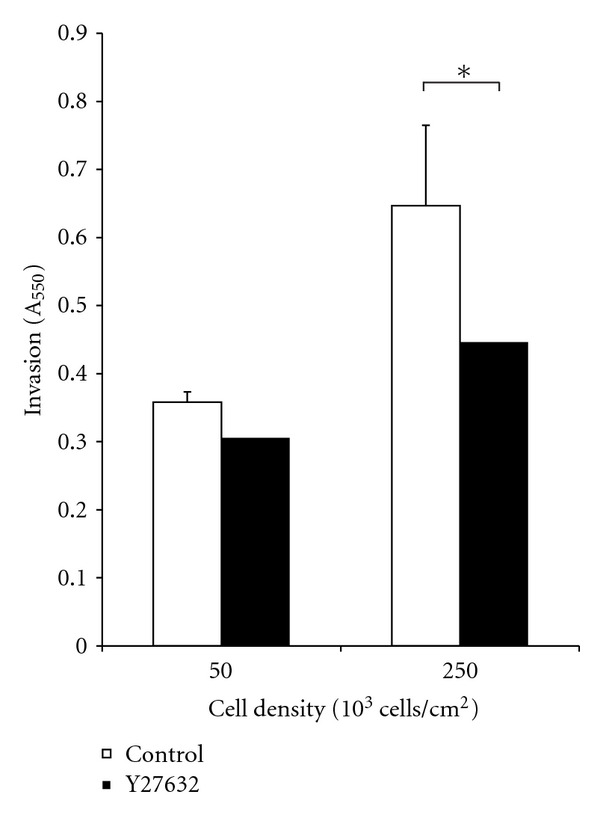
SW620 cells were seeded at densities of 50 × 10^3^ and 250 × 10^3^ cells/cm^2^ onto Boyden chambers with an 8 *μ*m pore membrane. The wells were treated with Y-27632 to study the impact of ROCK inhibition on cell invasion in a low- and high-density environment. A 3.5-fold increase in cell invasion was observed in Y-27632-treated cells seeded at a lower density of 50 × 10^3^ cells/cm^2^.

Cell invasion increased approximately 2-fold in samples with cell density of 250 × 10^3^ cells/cm^2^. However, it should be noted that the initial seeding condition for high cell-density sample was 5 times higher than the low seeding density of 50 × 10^3^ cells/cm^2^. The result that was most significant was that ROCK inhibition with Y-27632 decreased invasion in higher cell densities while it had minimal impact on invasion at lower seeding densities. Approximately, 1.5-fold decrease in cell invasion was observed in samples seeded at 250 × 10^3^ cells/cm^2^ and treated with Y-27632. Standard Student *t*-test was performed, and a *P* value of 0.05 was considered significant (∗). The data is shown in Figure 3.

### 3.4. Y-27632 Increases Invasion at Low Densities in Collagen Gels

**Figure 4:**
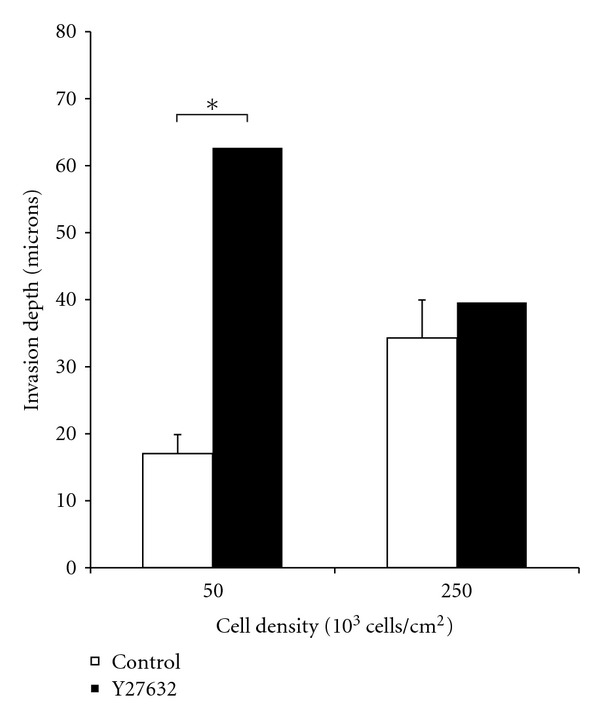
SW620 cells were seeded at densities of 50 × 10^3^ and 250 × 10^3^ cells/cm^2^ onto 1.5 mg/mL collagen I gels. Scaffolds were treated with Y-27632 to study the impact of ROCK inhibition on cell invasion in a low- and high-density environment. Treatment with Y-27632 resulted in a 3.5-fold increase in cell invasion for cells seeded at 50 × 10^3^ cells/cm^2^.

Samples were tracked using a cell tracker dye. Seeding density did affect invasion depth of SW620 cells in collagen I scaffolds. Cells seeded at 250 × 10^3^ cells/cm^2^ invaded twice the distance than those seeded at 50 × 10^3^ cells/cm^2^ for untreated. However, this effect was not observed for SW620 cells treated with Y-27632. For cells seeded at the lower seeding density, treatment with Y-27632 led to a 3.5-fold increase in invasion depth. However, no significant increase was observed in cells seeded at 250 × 10^3^ cells/cm^2^. Standard Student *t*-test was performed and a *P* value of 0.05 was considered significant (∗). The data is shown in Figure 4.

### 3.5. Increasing Cell Seeding Density Increases Proliferation

Cell density was altered by changing the area while keeping the overall number of cells constant. Cell seeding density impacted cell proliferation. Increasing the cell seeding density from 50 × 10^3^ cells/cm^2^ to 250 × 10^3^ cells/cm^2^ led to a 2.5-fold increase in proliferation for untreated cells. For cells treated with Y-27632, increasing cell seeding density led to a 1.5 increase in proliferation. An increase in proliferation was expected since it has been shown in literature that cell proliferation increases when seeded in high cell-density environments [22].

**Figure 5:**
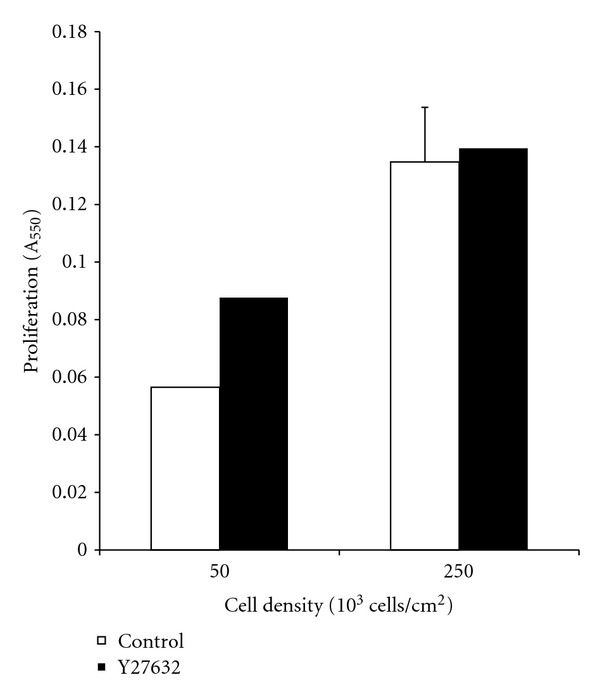
SW620 cells were seeded at densities of 50 × 10^3^ and 250 × 10^3^ cells/cm^2^ onto 1.5 mg/mL collagen I gels. Scaffolds were treated with Y-27632 to study the impact of ROCK-1 inhibition on cell proliferation in a low- and high-density environment. Treatment with Y-27632 resulted in a modest increase in cell proliferation for cells seeded at 50 × 10^3^ cells/cm^2^ and no significant change for those seeded at 250 × 10^3^ cells/cm^2^. Furthermore, increasing cell density increased cell proliferation 2.5-fold for the untreated condition and 1.5-fold for those treated with Y-27632.

For cells seeded at 50 × 10^3^ cells/cm^2^, treatment with Y-27632 led to a 1.5-fold increase in cell proliferation compared to that of the untreated samples. However, for cells seeded at 250 × 10^3^ cells/cm^2^, treatment with the ROCK inhibitor did not appear to have a significant impact on cell proliferation. This data is shown in Figure 5.

### 3.6. ROCK-I Knockdown Leads to Increased Invasion

**Figure 6:**
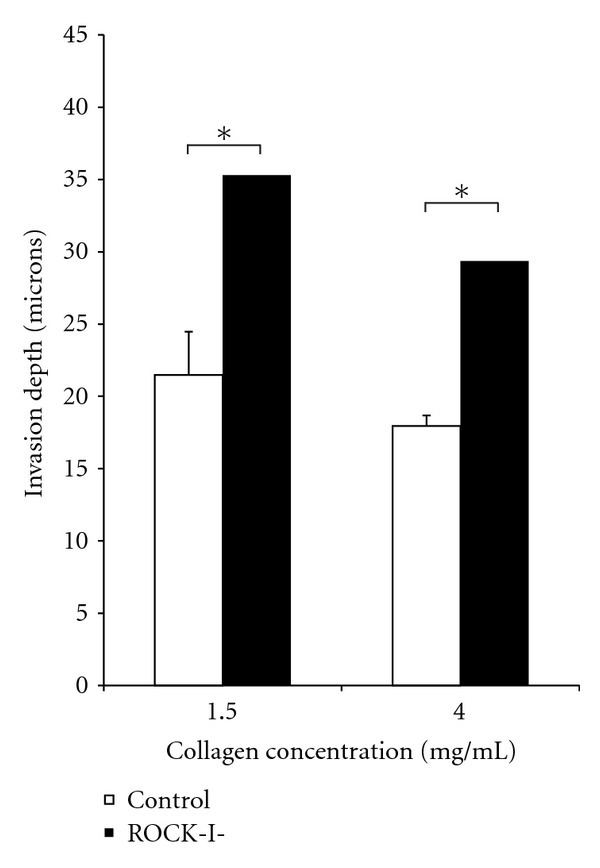
SW620 cells were seeded at densities 250 × 10^3^ cells/cm^2^ onto 1.5 and 4.0 mg/mL collagen I gels. ROCK-1 was knocked down via siRNA to study the impact of ROCK-I on cell invasion in a low- and high-density environment. ROCK-I knockdown resulted in a 60% increase in invasion for cells seeded in both 1.5 and 4.0 mg/mL scaffolds.

SW620 cells were transfected with siRNA as described in Materials and Methods. Samples were tracked using a cell tracker dye. Increasing collagen concentration did seem to reduce overall invasion, though the effect was slight. Cells seeded onto 4.0 mg/mL collagen I gels had a ~20% less invasion depth than those seeded onto 1.5 mg/mL gels for both ROCK-I knockdown as well as untransfected cells. For both 1.5 and 4.0 mg/mL collagen I gels, ROCK-I knockdown resulted in a 1.6-fold increase in invasion depth compared to untransfected cells. Standard Student *t*-test was performed, and a *P* value of 0.05 was considered significant (∗). This data is shown in Figure 6.

### 3.7. ROCK Knockdown Decreased Invasion for Dense Gels

SW620 cells were transfected with siRNA as described in methods section. Collagen concentration had a significant impact on proliferation of untransfected SW620 cells. Cells seeded onto 4 mg/mL collagen gel had a proliferation value that was 2.5-fold greater than that seen in cells seeded onto 1.5 mg/mL collagen gels. However, cells where ROCK-I was knocked down resulted in a 1.3-fold increase by increasing collagen concentration.

**Figure 7:**
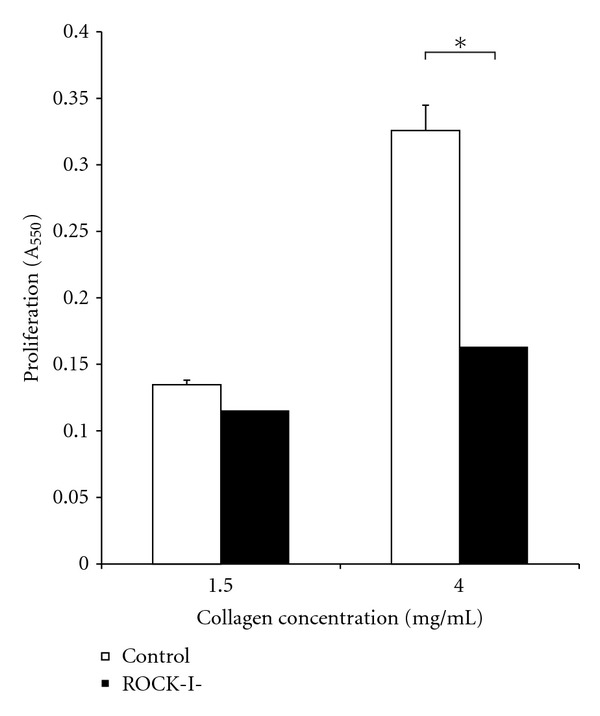
SW620 cells were seeded at densities 250 × 10^3^ cells/cm^2^ onto 1.5 and 4.0 mg/mL collagen I gels. ROCK-1 was knocked down via siRNA to study the impact of ROCK-I on cell invasion in a low- and high-density environment. Increasing collagen density resulted in a 2.5-fold increase in cell proliferation for the untransfected condition but only a 1.6-fold increase for those where ROCK-I expression was silenced. ROCK-I knockdown resulted in a 50% decrease in cell proliferation in cells in 4.0 mg/mL collagen I scaffolds.

 There was no significant difference between ROCK-I knockdown and ROCK-II knockdown with respect to cell proliferation in either of the collagen concentrations (Data not shown). However, ROCK knockdown had differing impacts in the collagen gels of differing concentrations. ROCK-I knockdown led to an insignificant decrease in cell proliferation in 1.5 mg/mL gels. However, for cells in collagen gels of 4.0 mg/mL, proliferation was cut by 50% due to ROCK knockdown. Standard Student *t*-test was performed and a *P* value of 0.05 was considered significant (∗). This data is shown in Figure 7.

## 4. Discussion

Cancer remains one of the leading causes of mortality world-wide with colon cancer being the third most commonly diagnosed cancer and the third leading cause of cancer death in both men and women in the USA [1, 2]. Rho-kinase is known to be an important pharmacological target for cancer because of its role in the invasion and migration of cancer cells [23, 24]. We have previously linked ROCK-II knockdown with decreased invasion in SW620 cells in prior studies [20]. In this study, our goal was to analyze the effect of ROCK-I inhibition on colon cancer cell invasion. Although Y-27632 targets both ROCK isoforms, it is a more potent inhibitor of ROCK-I than ROCK-II [19].

The most surprising, yet revealing data comes from studying invasion depth of varying densities of SW620 cells in 3D collagen I scaffolds. When seeded at a low cell density, ROCK inhibition by Y-27632 resulted in a dramatic increase in invasion. However, as cell density increased, this effect was attenuated. This indicates that cell density plays a major role in governing how the ROCK inhibitors affect the pro-invasiveness of SW620 cells.

 Other studies have shown that treatment with Y-27632 led to decreased metastasis, especially in breast cancer both *in vitro* and *in vivo *[25]. Also, it has been suggested by others that Y-27632 decreased the expression of LIMC and MLC suggesting inhibition of metastasis [26] and decreased invasion in a meningitis model [27]. Treatment with ROCK inhibitors, especially Y-27632, has also been shown to initiate loss of stress fibers and a concomitant decrease in tyrosine phosphorylation of paxillin and Focal Adhesion Kinase (FAK) [28] thus reducing cell-ECM contact. While literature suggests treatment with Y-27632 should decrease invasion, our 3D invasion data contradicts this viewpoint. Therefore, we conducted more traditional invasion assays based on boyden chambers and found that treatment with the inhibitor can reduce invasion in these assays. So, the discrepancy in effect of treatment with Y-27632 can be linked to the microenvironment of the seeding conditions. Since we have previously linked ROCK-II knockdown to decreased invasion in similar 3D collagen microenvironments, we hypothesized that it is ROCK-I knockdown which leads to increased invasion in 3D collagen I scaffolds. ROCK-I has been linked to facilitate stress fiber formation and promote focal adhesions in fibroblasts [29]. Another study has shown that ROCK-I knockdown led to decreased adhesion in keratinocytes on fibronectin [18]. This loss of adhesion may in fact facilitate cell motility thereby aiding in increased cell invasion. To test the role of ROCK-I inhibition on invasion, we knocked down ROCK-I by treating SW620 cells with Y-27632 in scaffolds of varying collagen concentrations and found that ROCK-I knockdown resulted in increased invasion in both collagen concentrations. 3D collagen scaffolds mimic the *in vivo* microenvironment of a colon tissue better than the Boyden chamber assembly, since 3D collagen matrix has comparable tissue density as the *in vivo* environment. Thus, the increase in invasion of SW620 cells when seeded on 3D collagen matrix and treated with Y-27632 suggests that treatment with ROCK-I inhibitor may in fact be detrimental in the event of colon cancer in an *in vivo* model.

In summary, we demonstrate that ROCK-I inhibition results in increased invasion in a three-dimensional collagen I model. While this may seem to contrast results of other studies, it is important to understand the significance of the microenvironment on the results. Two-dimensional substrates have fewer points and directions for cell attachment compared to a 3D collagen matrix. Therefore, the reduction of focal adhesions due to ROCK-I knockdown could and likely does have differing effects on cells in these varying environments. Thus suggesting that in an *in vitro* 3D collagen matrix, which resembles an *in vivo* model, treatment with Y-27632 may further increase cell invasion. However, further studies would have to be conducted assessing the impact of ROCK-I on cell-substrate attachment in both 2D and 3D environments as well as the effect of Y-27632 in an *in vivo* cancer model.
